# Utilization of Magnetic Resonance Imaging in Research Involving Animal Models of Fetal Alcohol Spectrum Disorders

**DOI:** 10.35946/arcr.v37.1.04

**Published:** 2015

**Authors:** Xiaojie Wang, Christopher D. Kroenke

**Affiliations:** Xiaojie Wang, Ph.D., is a postdoctoral fellow in the division of neuroscience, Oregon National Primate Center, at Oregon Health & Science University, Portland, Oregon.; Christopher D. Kroenke, Ph.D., is an associate scientist in the division of neuroscience, Oregon National Primate Center, and an associate professor in the Advanced Imaging Research Center and department of behavioral neuroscience at Oregon Health & Science University, Portland, Oregon.

**Keywords:** Fetal alcohol exposure, prenatal alcohol exposure, fetal alcohol spectrum disorder, fetal alcohol effects, brain, developing brain, fetal development, central nervous system, magnetic resonance imaging, neuroimaging, animal models

## Abstract

It is well recognized that fetal alcohol exposure can profoundly damage the developing brain. The term fetal alcohol spectrum disorder (FASD) describes the range of deficits that result from prenatal alcohol exposure. Over the past two decades, researchers have used magnetic resonance imaging (MRI) as a noninvasive technique to characterize anatomical, physiological, and metabolic changes in the human brain that are part of FASD. As using animal models can circumvent many of the complications inherent to human studies, researchers have established and explored a number of models involving a range of species. Using MRI-based modalities, the FASD animal models have demonstrated decreased brain volume and abnormal brain shape, disrupted cellular morphology differentiation, altered neurochemistry, and blood perfusion. These animal studies have facilitated characterization of the direct effects of ethanol; in many cases identifying specific sequelae related to the timing and dose of exposure. Further, as a result of the ability to perform traditional (such as histological) analyses on animal brains following neuroimaging experiments, this work leads to improvements in the accuracy of our interpretations of neuroimaging findings in human studies.

Neuroimaging, particularly magnetic resonance imaging (MRI), has begun to tease apart the underlying mechanisms behind alcohol’s deleterious effects on the fetus and eventually may lead to earlier detection of what can be devastating child neurodevelopmental deficits. In 1968, researchers first reported an association between prenatal alcohol exposure and what can be persistent adverse cognitive, behavioral, motor, and psychosocial outcomes, leading to the first description of fetal alcohol syndrome (FAS) (Jones and Smith 1973). FAS, as described by prenatal and/or postnatal growth retardation, central nervous system (CNS) involvement, and facial dysmorphology, represents some of the most extreme effects of maternal alcohol use. However, there is a broader spectrum of symptoms, with some individuals prenatally exposed to alcohol having significant neurobehavioral deficits but not the full FAS symptomology (Mattson et al. 1997). To better represent the effect of alcohol on children prenatally exposed to alcohol, clinicians and researchers now use the term fetal alcohol spectrum disorder (FASD) (Mattson et al. 1998).

Although researchers have established a causal relationship between fetal alcohol exposure and life-long cognitive and behavioral impairment, it remains less clear how changes in the developing brain mediate these impairments. In addition, the detection of FASD remains elusive as the diagnostic criteria of FAS/FASD typically only allow for identification of affected individuals in late childhood. Noninvasive neuroimaging techniques hold potential for both identifying the underlying mechanisms behind alcohol’s deleterious effects on the central nervous system (CNS) and helping detect FAS/FASD much earlier. And although studies in humans have provided some insight into these issues, studies in animals allow researchers to ask far more detailed questions.

## MRI Techniques in Humans

MRI is a safe, noninvasive neuroimaging method that allows repetitive examination of human brains. It provides relatively high spatial resolution (approximately 1 × 1 × 1 mm^3^ in most modalities) and a rich toolbox that enables researchers to perform anatomical, physiological, and metabolic measurements (see sidebar on “Magnetic Resonance Imaging Techniques” for detailed descriptions of the various techniques). Over the past two decades, various MRI techniques have uncovered brain abnormalities that are associated with cognitive/behavioral deficits in FASD-affected individuals (Riley et al. 1995, 2004).

**Magnetic Resonance Imaging [MRI] Techniques**

To produce image contrast, conventional MRI utilizes the fact that water 1H nuclei of different tissue types have different T1 and T2 relaxation times. By varying data acquisition parameters such as time of repetition (TR) and/or time of echo (TE), contrast can be tuned to enhance differentially anatomical structures such as gray matter, white matter, and cerebrospinal fluid (CSF). As a result, imaging can segment specific anatomical structures such as basil ganglia, cerebellum, corpus callosum, and hippocampus to facilitate quantitative volume and shape analyses (for the definition of these and other terms, see Glossary).

**Diffusion Tensor Imaging (DTI)**

Water diffuses through biological tissues based on thermally driven Brownian motion and is impeded by myriad structures. During the typical diffusion time in a diffusion magnetic resonance (MR) scan (10 to 100 ms), the behavior of water diffusion within the central nervous system (CNS) can vary dramatically depending on the tissue subtype. In cerebrospinal fluid, water experiences free and isotropic diffusion, which means it moves equally in all directions. In mature white matter and some gray matter regions (e.g. hippocampus, cerebellum, and cerebral cortex), interactions with biological membranes significantly reduces water diffusion perpendicular to dominant cellular processes (axons, dendrites, and glial processes). Thus, the diffusion is direction dependent, or what is known as anisotropic. In gray matter regions that lack highly oriented cellular structures, water molecules experience boundaries in a more random fashion and this situation often is referred to as restricted isotropic diffusion.

**Figure:**
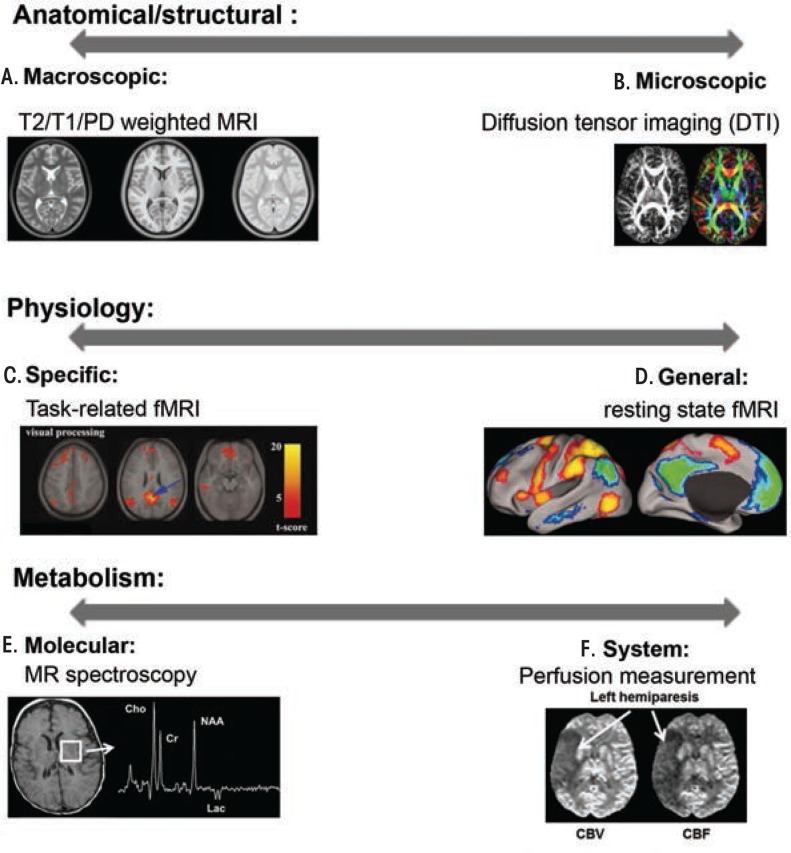
SOURCES: C. Greicius et al. 2003; D. Fox et al. 2005: E. Gujar et al. 2005; F. Calamante et al. 1999

For each imaging voxel, DTI measurements can derive multiple parameters. One commonly used parameter is fractional anisotropy (FA), which characterizes the degree of anisotropy of a diffusion process. FA measurements range between 0, which represents isotropic diffusion as in free water or cerebrospinal fluid (CSF), and 1, which indicates that diffusion is completely restricted along one or more directions. Therefore, high FA reflects coherent and highly orientated fiber tracts and decreased FA often indicates myelin and axon injury, and/or any disruption of fiber tracts. Mean diffusivity (MD) is a scalar measure of the total diffusion within a voxel and reflects the mobility of water molecules. MD is generally high in CSF, and lower in normal gray and white matter. Compared with anatomical MRI, DTI-derived metrics are more sensitive to the changes on a cellular level (Mori and Zhang 2006).

**Functional MRI (fMRI)**

fMRI is a technique that measures brain physiological activity. It does so based on the coupling of blood flow and neuron activity and the difference in water 1H spin relaxation between environments of deoxyhemoglobin and oxyhemoglobin.

In typical task-based fMRI experiments (Logothetis 2008), a subject alternates between a specific task-responding state and a control state. In brain areas where a task activates neurons, blood flow is altered such that more oxyhemoglobin is present compared to deoxyhemoglobin. This results in a transient task-dependent increase in magnetic resonance (MR) signal intensity within the brain regions that the task activates and this phenomenon is termed blood oxygen level dependent (BOLD) MR signal.

In resting-state fMRI experiments (Fox et al. 2005), no stimulus is presented to the subject, and temporally correlated MR intensity fluctuations are used to infer disparate brain areas that are functionally related to each other.

**MR Spectroscopy (MRS)**

MR spectroscopy provides a quantitative and specific measure of brain chemistry. While conventional MRI primarily detects water 1H nuclei, MRS detects proton signals from other molecules such as amino acids (e.g. glutamate), lipids, lactate, N-acetylaspartate (NAA), choline, creatine, and, when present, ethanol. Alternatively, MRS can also detect other MR active nuclei (e.g. 31P, 23Na, 19F, etc). Researchers can use MRI and MRS in combination: MRI to identify an anatomical location and localized MRS to detect the concentration of specific metabolites within the region of interest. Among the MRS studies of human and animal models of FASD, researchers most frequently examine NAA, choline-containing compounds (Cho), and creatine/phosphocreatine (Cr) signals. The NAA signal includes contributions from primarily NAA, and to a lesser extent from N-acetylaspartylglutamate (NAAG). NAA is considered to be a marker that reflects neuronal/axonal health, viability and density. Cho signals consist of multiple choline derivatives which are precursors or degradation products of the membrane phospholipids. Thus, the Cho signal is seen as a marker for cell membrane integrity and myelination. The Cr signal, constituted of both creatine and phosphocreatine, is thought to reflect energy phosphate metabolism. As the absolute measurements of signal intensities of these metabolites are subject to source errors including CSF contamination, the Cr peak, which is relatively constant between individuals and most brain areas, is often used as internal reference. Thus in this review, we only discuss the NAA/Cr and Cho/Cr ratios (Graaf 2002).

**MR Perfusion Measurements**

Cerebral blood flow (CBF), is the blood supply to the brain at any given time and is tightly regulated to meet the brain’s metabolic demands. In an adult, CBF is typically 750 ml/min. This equates to an average perfusion of 50–54 ml of blood per 100 g of brain tissue per minute. A number of MR modalities can be used to measure blood perfusion within the brain, such as dynamic susceptibility contrast (DSC) MRI, dynamic contrast enhanced (DCE) MRI, and arterial spin labeling (ASL).

DSC-MRI involves the injection of a bolus paramagnetic contrast agent. Then a fast imaging sequence is used to acquire a series of T2^*^-weighted images during the contrast agent’s first passage through the tissue. The passage of the contrast agent leads to MR signal intensity drop due to the magnetic susceptibility effect. The signal intensity-time curve measured by the series of T2^*^-weighted images can be mathematically converted to a contrast agent concentration-time curve. The concentration-time curve is then integrated to give an index that is proportional to the relative cerebral blood volume (rCBV) of a given imaging voxel. Additionally, if such measurement is done within or near a major artery, the arterial input function can then be derived and in turn, relative cerebral blood flow can also be calculated (Calamante 1999).

ReferencesCalamanteFMeasuring cerebral blood flow using magnetic resonance imaging techniquesJournal of Cerebral Blood Flow and Metabolism19770173519991041302610.1097/00004647-199907000-00001de GraafRAIn Vivo NMR Spectroscopy: Principles and TechniquesNew YorkJohn Wiley & Sons2002FoxMDSnyderAZVincentJLThe human brain is intrinsically organized into dynamic, anticorrelated functional networksProceedings of the National Academy of Science of the United States of America102279673967820051597602010.1073/pnas.0504136102PMC1157105GujarSKMaheshwariSBjörkman-BurtscherISundgrenPCMagnetic resonance spectroscopyJournal of Neuroophthalmology25321722620051614863310.1097/01.wno.0000177307.21081.81GreiciusMDKrasnowBReissALMenonVFunctional connectivity in the resting brain: a network analysis of the default mode hypothesisProceedings of the National Academy of Science of the United States of America100125325820031250619410.1073/pnas.0135058100PMC140943LogothetisNKWhat we can do and what we cannot do with fMRINature45386987820081854806410.1038/nature06976MoriSZhangJPrinciples of diffusion tensor imaging and its applications to basic neuroscience researchNeuron51552753920061695015210.1016/j.neuron.2006.08.012OstergaardLSorensenAGKwongKKHigh resolution measurement of cerebral blood flow using intravascular tracer bolus passages. Part II: Experimental comparison and preliminary resultsMagnetic Resonance in Medicine3657267361996891602310.1002/mrm.1910360511

### Anatomical Differences

Traditional MRI studies show anatomical differences between the brains of children and adolescents with FASD and those not exposed to alcohol in utero, including the following:

- Significant reductions in overall brain volumes in children and adolescents with FASD (Archibald et al. 2001; Johnson et al. 1996; Lebel et al. 2008; Sowell et al. 2002; Willoughby et al. 2008);
- Reduced volumes in specific regions, including the caudate nucleus (Archibald et al. 2001; Cortese et al. 2006), hippocampus (Willoughby et al. 2008), and cerebellar vermis (Autti-Ramo et al. 2002); and
- Corpus callosum malformations in FASD individuals (Autti-Ramo et al. 2002; Bookstein et al. 2002; Johnson et al. 1996).

Study results are mixed regarding the effect of maternal ethanol exposure on fetal cerebral cortical thickness: Sowell and colleagues (2002, 2008) observed greater cortical thickness in parietal and posterior temporal regions, whereas Zhou and colleagues (2011) reported thinner cortical gray matter in a number of brain regions in an FASD group. Meanwhile, results from studies using diffusion tensor imaging (DTI), which allows researchers to assess tissue abnormalities on a microstructural level, even in the absence of gross dysmorphology, suggest that individuals exposed to alcohol in utero have less organized white matter fiber tracts (Wozniak and Muetzel 2011). Specifically, DTI showed significantly decreased fractional anisotropy and/or increased mean diffusivity (MD) in the corpus callosum (Ma et al. 2005) and other white matter regions (Fryer et al. 2009; Lebel et al. 2008; Sowell et al. 2008) of alcohol-exposed individual compared with those who were not exposed.

### Neurochemical Changes

Another technique, magnetic resonance spectroscopy (MRS) (see sidebar on “Magnetic Resonance Imaging Techniques”), offers a unique way to detect neurochemical changes by monitoring the concentration of neurometabolites, including choline- containing compounds (Cho), which are markers of cell membrane stability and myelination; N-acetylaspartate (NAA), a marker of neuronal/axonal viability and or density; and creatine/phosphocreatine (Cr), a marker of metabolic activity (Moffett et al. 2007). One MRS study (Fagerlund et al. 2006) comparing people with FAS with normal control subjects reported lower NAA levels in various brain regions among the FAS subjects, whereas another study (Cortese et al. 2006) found higher NAA levels in the caudate nucleus.

### Brain Activation Patterns

Functional MRI allows researchers to detect differences in brain activation patterns between FASD individuals and control subjects during various tasks involving spatial, verbal, and visual working memory (Astley et al. 2009; Malisza et al. 2005; O’Hare et al. 2009), verbal learning (Sowell et al. 2007), and inhibitory control (Fryer et al. 2007). These altered brain activation patterns might underlie the poor executive functioning-based skills observed in FASD individuals (Astley et al. 2009; Fryer et al. 2007; Malisza et al. 2005; O’Hare et al. 2009; Sowell et al. 2007).

### Drawbacks of Human Studies

Although neuroimaging and neuropathological investigations of the brains of FASD-affected individuals have elucidated specific abnormalities in brain structure, metabolism, and function underlying cognitive and behavioral impairments, studies of human subjects have a number of limitations. These include (1) the paucity of autopsy reports from children with FASD hampers interpretation of in vivo human neuroimaging data; (2) the related inability to evaluate and validate correlative structural and functional damage; (3) the difficulty in controlling, or even determining, variables such as dosing, timing, and consumption pattern of maternal drinking; and (4) the difficulty in eliminating confounds in human studies including environment, other maternal substance abuse, stress, and malnutrition. Animal models of FAS/FASD circumvent many of these inherent complications.

## MRI in Animals

**Figure 1:**
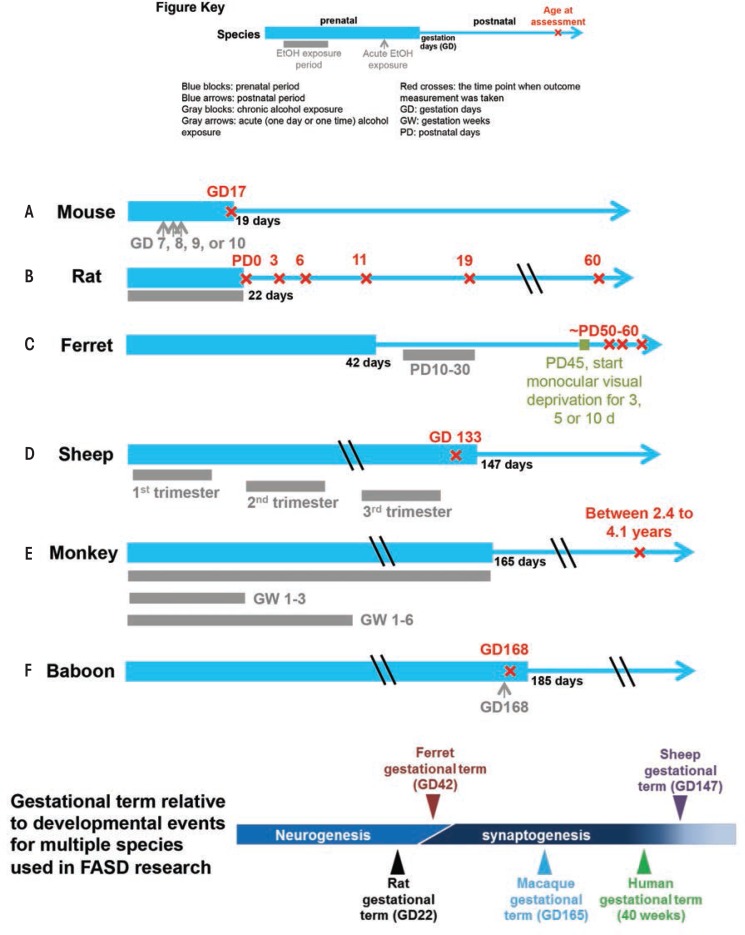
Timing schemes of popular animal models for FASD research.

Animal models allow researchers to control maternal and environmental variables such as genetic background, nutritional status, dosage, and timing pattern of ethanol insult, which frequently enables experiments to focus on the mechanisms of ethanol’s teratogenic action. As early as 1977, studies of mouse and rat FASD models confirmed the causal relationship between prenatal alcohol exposure and FASD, which had been speculated in clinical observations (Abel and Dintcheff 1978; Chernoff 1977). Shortly after, Sulik and colleagues (1981) demonstrated that treating pregnant mice with alcohol at gestation day (GD) 7 (equivalent to human gestation week [GW] 3) resulted in facial dysmorphology in their offspring, a finding consistent with FASD-affected human infants. Since then, researchers have established a number of animal models in a range of species to study the mechanism of alcohol’s teratogenic effects, to test the efficacy of protective interventions, and to improve the sensitivity and specificity of neuroimaging techniques for identifying FASD (see figure 1). Each model provides certain advantages and disadvantages as described below.

### Nonmammalian Animals

Nonmammalian animal models, including zebra fish, fruit fly, and frog, offer unique advantages by providing flexible and well-characterized experimental systems. However, more phylogenetically advanced vertebrate species are necessary in studies that require a complex CNS and long developmental periods.

### Rodents

Among mammalian species with a more complex CNS, rodents are a very important model system. They have a short reproductive cycle, their genetic background is readily controlled, and their small size makes them suitable for smallbore animal MRI systems.

### Ferrets

Ferrets also have a fairly short pregnancy period relative to CNS developmental milestones (see figure 1C) and, as a result, are advantageous for investigating early neurodevelopmental disruption without the complication of needing to induce premature delivery, or perform in utero manipulations.

### Sheep

Sheep have a long gestational term relative to CNS development, more resembling human gestation, which allows investigators to explore various drinking patterns and exposure times more similar to those seen in humans.

### Nonhuman Primates

The CNS develops very similarly in nonhuman primates and humans. In addition to their large and highly folded brains, nonhuman primates also exhibit more complex social relationships and cognitive functions. Thus, they can serve as a bridge between studies in other animal models and humans (Miranda-Dominguez et al. 2014). In a number of cases, studies conducted on FASD animal models have productively used MRI as a noninvasive neuroimaging modality. The primary outcomes from studies in all of these models, which are reviewed below, are summarized in the table.

## Findings From Animal Models

### Rodents

**Figure 2:**
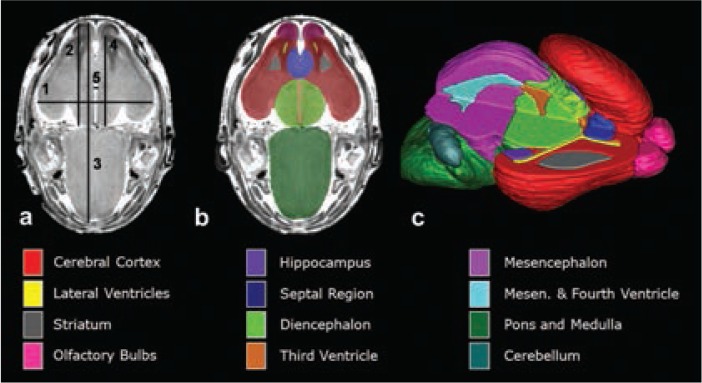
High-resolution magnetic resonance (MR) images of mouse fetuses at gestational day (GD) 17 allow for linear measurements, regional segmentation, and three-dimensional reconstruction. **(A)** A horizontal image with lines depicting sites of linear measurement as follows: brain width (biparietal distance), line 1; bulbothalamic distance, line 2; mid-sagittal brain length, line 3; frontothalamic distance, line 4; third ventricle width, line 5. (Cerebellar width [transverse cerebellar distance, not included] was measured at its greatest dimension.) Manual segmentation, as depicted by the color-coded regions in **(B)** allowed for subsequent three-dimensional reconstruction **(C)** and analyses of selected brain regions. **(C)** The upper right quadrant of the brain has been removed to allow for visualization of the interior structures. Color codes for the segmented brain regions shown are at the bottom of the figure. NOTE: Figure adapted from (Godin et al. 2010).

**Figure 3:**
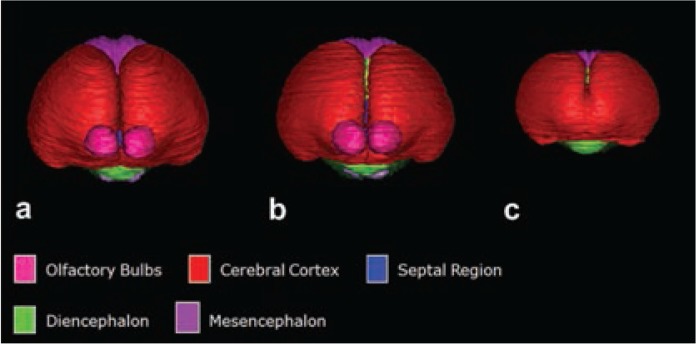
Reconstructed brains of a control fetal mouse at gestational age 17 **(A)** along with the brains of ethanol-exposed fetuses having mid-facial abnormality **(B** and **C)**. Segmented magnetic resonance microscopy scans of control **(A)** and ethanol-exposed **(B** and **C)** fetuses were reconstructed to yield whole brain (frontal view). Although the affected fetus in **(B)** had a normal-appearing face (figure not shown here), a slight widening of the space between the cerebral hemispheres (as evidenced by visibility of the septal region and diencephalon) can be seen as compared with control **(A)**. Missing olfactory bulb and rostral union of the cerebral hemispheres can be seen in fetus **(C)**. NOTE: Figure adapted from (Godin et al. 2010).

In a series of mouse FASD studies, researchers used ex vivo MRI to examine the effect of acute ethanol insult on GD 7, 8, 9, and 10, a time range that corresponds to human GWs 3 to 4 (Godin et al. 2010; O’Leary-Moore et al. 2010; Parnell et al. 2009, 2013). To characterize ethanol-induced structural brain abnormalities, they analyzed high-resolution MR images of each fetus dissected on GD 17 (see figure 2). They measured key growth metrics such as brain width, mid-sagittal brain length, and third ventricle width in a single image plane (see figure 2A). They also segmented and then reconstructed regional brain structures (e.g. cerebral cortex, ventricles, cerebellum, etc.) to quantify their volume and morphology in three dimension (see figure 2B and C). In the fetuses exposed to ethanol in utero, the researchers found notable volume reductions across various brain regions, which were accompanied by increased ventricular sizes. They also observed regional brain morphology changes including holoprosencephaly, or the absence of midline cerebral structures, and widened space between cerebral hemispheres (see figure 3B and C). These results demonstrate that an acute maternal alcohol insult on GD 7 to 10 leads to a spectrum of forebrain deficiencies in mouse fetuses. Importantly, some animals that exhibited CNS malformations did not have facial dysmorphology. This series of studies employing an acute, high-dose maternal ethanol treatment paradigm helped titrate sensitive periods for a variety of malformations and extended our knowledge of the dependency of ethanol teratogenesis on the timing of exposure during gestation.

A more recent study conducted by the same research group examined brain dysmorphology resulting from maternal dietary ethanol intake at a much lower dose than the previous study and occurring during the time period equivalent to the first trimester in humans (Parnell et al. 2014). Using the same MRI-based volumetric measurements, the researchers observed reduced cerebellum and enlarged septal region in a GD 7 to 11 ethanol-exposure group. In a GD 12 to 16 ethanol-exposure group, the researchers detected size reductions in right hippocampus and increased pituitary gland volume. Overall, the number of brain regions significantly affected and the severity of the effect were less than those following acute, high-dose exposures. The application of high-resolution MRI here has facilitated the systematic and comprehensive examination of the brain abnormalities caused by prenatal ethanol exposure. The employment of a mouse FASD model in these studies allowed the control of variables, especially ethanol exposure patterns, which, in turn, aided in confirming that the type and severity of ethanol-induced birth defects largely depend on the treatment pattern and dosage along with the developmental stage at the time of ethanol exposure (Godin et al. 2010; O’Leary-Moore et al. 2010; Parnell et al. 2009, 2013, 2014).

A study in rats using ex vivo high-resolution MRS examined regional neurochemistry in frontal cortex, striatum, hippocampus, and cerebellum in postnatal day [PD] 16 animals exposed to ethanol as neonates (PD 4 to 9) (O’Leary-Moore et al. 2008). The technique allowed them to measure the relative concentrations of certain brain metabolites, comparing the brains of ethanol exposed rats with those of control rats. They found changes in several metabolites in various brain regions:

- The NAA/Cr ratio was reduced in the cerebellum, which likely reflects delayed development, cell loss, or both in these regions. This finding supports those of a human FASD study (Fagerlund et al. 2006), reporting a decrease in NAA/Cr in cerebellum along with other brain regions. That said, another human FASD study (Cortese et al. 2006) reported an increased NAA/Cr ratio in the caudate nucleus in FASD individuals. The researchers suggested that this increase might be indicative of a “lack of normal programmed cell death, dendritic pruning/myelination during development” (p. 597).
- The Cho/Cr ratio was significantly lowered in hippocampus and elevated in striatum in ethanol-exposed rats compared with controls. The researchers concluded that these changes in Cho level were “consistent with dysfunctional membrane turnover in the young perinatal ethanol-exposed brain” (p. 1704).
- The concentration of the amino acid taurine was reduced in hippocampus and striatum. Taurine deficits can cause growth retardation and impaired CNS function (Aerts and Van Assche 2002).
- Glutamate, an excitatory neurotransmitter, was reduced in cerebellum only from prenatal ethanol-exposed female rats, indicating disrupted glutamatergic function (O’Leary-Moore et al. 2008).
- A trend of decreased γ-aminobutyric acid (GABA) (without statistical significance) also was observed in the striatum and cerebellum in the rats with neonatal ethanol exposure.

**Figure 4:**
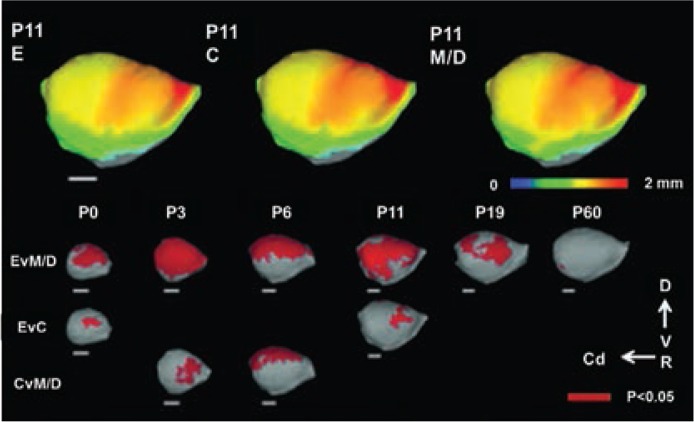
Regional pattern of cerebral cortical thickness differences result from threshold-free cluster enhancement (TFCE) analysis. On the top row, mean cortical thickness at postnatal day (PD) 11 for each group in the rat (*n* = 4 to 6/age/group) are projected onto target cortical surfaces. TFCE results are pictured in dark red in the last three rows representing regions in which mean cortical thickness between groups is significantly different (*P* < 0.05). Specific regional differences, centered on primary sensory areas were found among ethanol **(E)** and maltose/dextrin (M/D) groups at all ages. Regions of significant difference also were found in comparisons between E and control **(C)** groups at PD 0 and PD 11 and between control **(C)** and M/D groups at P 3 and P 6. Scale bars (in white) represent 2 mm. D, dorsal; V, ventral; Cd, caudal; R, rostral. NOTE: Figure adapted from Leigland et al. 2013*a*.

The ability to study this broad range of MRS signals in animal models may hold potential for the development of additional biomarkers for FASD diagnosis and treatment evaluation. In another rat study (Leigland et al. 2013*a*), researchers used ex vivo MRI to examine the cerebral cortex of rat pups born to dams treated with ethanol throughout gestation, comparing them with pups whose moms either received maltose/dextrin instead of ethanol or no treatment. They performed cross-sectional measurements on the pups on PD 0, 3, 6, 11, 19, and 60 (see figure 1B). The ethanol-exposed pups had reductions in volume, thickness, and surface area of the cerebral cortex on PD 0, compared with control and M/D-treated groups, and the difference persisted into adulthood (PD 60). To examine whether prenatal ethanol exposure differently affected particular areas of the cerebral cortex, the researchers analyzed differences in regional patterns of cortical thickness. They saw a significant difference in the parietal and frontal-parietal region of the cortex or somatosensory and motor locations (see figure 4). This finding agrees with a human study observing smaller cortical thickness in FASD (Zhou et al. 2011) but is at odds with reports by Sowell and colleagues (2002, 2008, 2001), in which greater cortical thickness was reported in FASD-affected individuals. The discrepancy between the two human studies might be explained by differences in the image processing procedures used. Leigland and colleagues (2013*a*), in the rat study, and Zhou and colleagues (2011), in the human study, recorded absolute cerebral cortical thickness/volume; Sowell and colleagues (2001) normalized individual gray matter volume to total brain volume before statistical analyses in their study. If fetal ethanol exposure disproportionately affects shrinkage of different brain structures, it is possible that differences in the direction of effect on cerebral cortical thickness could result from the different data processing strategies.

**Figure 5:**
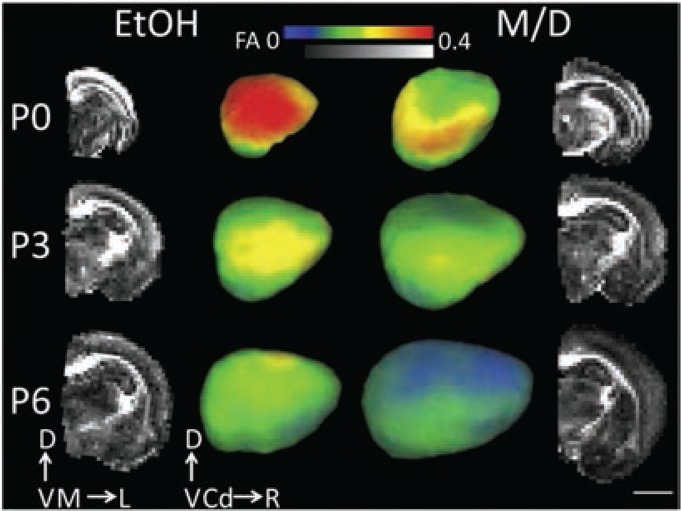
Effect of prenatal ethanol exposure on cerebral cortical fractional anisotropy. The two middle columns of images are laterally facing mid-cortical surface models of one rat at PD 0, PD 3, and PD 6 right hemisphere for each treatment group (ethanol) and maltose/dextrin (M/D), on which cortical fractional anisotropy (FA) at each mid-cortical surface node is projected. The outer columns represent mid-coronal FA maps for the right hemisphere of the same subjects depicted in the middle columns. Cortical FA decreased significantly with age. Additionally, cortical FA was largest, and isocortical volume smallest, in the ethanol group compared with the M/D group. This group difference is most visible in the outer layers of the cortex. NOTE: Scale bar is 4 mm. D = dorsal, V = ventral, M = medial, L = lateral, Cd = caudal, R = rostral. Figure adapted from Leigland et al. 2013*b*.

Although a majority of neuroimaging research on early cerebral cortical development has focused on gross volume change and dysmorphology, one study used ex vivo DTI on rats to characterize prenatal ethanol exposure’s effect on cortical neuron morphological differentiation (Leigland et al. 2013*b*). Rats exposed to daily ethanol throughout gestation exhibited a higher diffusion fractional anisotropy (FA) in their cerebral cortex compared with age-matched M/D controls at ages PD 0, PD 3, and PD 6, indicating a higher preference for water to diffuse radially rather than parallel to the pial surface (figure 5) (see sidebar “Magnetic Resonance Imaging Techniques” for explanation of the technique). The researchers validated this finding with quantitative histological analyses of the same brains. They found that higher FA reflected a more simple and coherent cortical cellular structure, which has previously been shown with traditional invasive anatomical measurement methods (Cui et al. 2010; Davies and Smith 1981; Fabregues et al. 1985; Hammer and Scheibel 1981) to result from ethanol-induced disruption in neuronal differentiation. The framework proposed in this study in which cellular-level microstructure can be inferred by DTI-derived FA provides a novel strategy for characterizing the effects of ethanol exposure on cerebral cortical gray matter.

### Sheep and Ferret

Although, to our knowledge, no MRI studies have been published on fetal alcohol exposed sheep or ferrets, we review here some results using these species for FASD research. Gestational term lengths in these species, relative to other developmental events, represent extremes, and these properties have been exploited to address specific scientific questions. Similar to humans, sheep have a long gestation time and all three trimester equivalents occur in utero. Studies have found that binge ethanol exposure in all three trimesters leads to deficits in fetal cerebellar Purkinje cells (Ramadoss et al. 2007*a*,*b*) (see figure 1D). Another study using a sheep FASD model reported that second-trimester alcohol exposure has an adverse effect on fetal cerebral blood flow (Mayock et al. 2007). In contrast to sheep, ferrets have a short gestation time relative to CNS development, and its third-trimester equivalent of human gestation occurs postnatally. During this time, exposure to ethanol can disrupt neuronal differentiation, synaptogenesis, circuit formation, and remodeling of neuronal connections. Medina and colleagues (2003) have used a ferret monocular deprivation model, a well-characterized model of neuronal plasticity in the neocortex, to find that a 3-week alcohol exposure starting PD 10 impairs ocular dominance plasticity at a later age (see figure 1C), indicating ethanol insult during this time could have a profound effect on development and plasticity of neural circuits in the neocortex.

### Nonhuman Primates

As early as 1995, Astley and colleagues used MRI and MRS to study brain structural and biochemical changes in a macaque monkey model of FASD (Astley et al. 1995). In this study, they explored three ethanol exposure patterns: once per week throughout the entire gestation period, once per week through GW 1 to 3, and once per week through GW 1 to 6 (see figure 1E). The researchers conducted MRI and MRS on the offspring of these treated monkeys between ages 2.4 and 4.1 years. Radiologists blinded to the monkeys’ alcohol exposure inspected the MRI images and found no difference in morphology or size of cerebral hemispheres, corpus callosum, brain stem, or cerebellum. However, MRS from the thalamus, parts of the internal capsule, and basal ganglia detected a significant increase in Cho/Cr ratio with increasing duration of in utero ethanol exposure. Importantly, the study also found the Cho/Cr ratio to be associated with increased cognitive impairment as assessed by the Infant Development Impairment Score. Further analyses of NAA/Cr and NAA/Cho ratios suggested that the Cho component changes with increasing ethanol exposure time. The researchers speculated that higher choline content might be associated with membrane breakdown.

A more recent study used dynamic susceptibility contrast (DSC)-MRI (see sidebar “Magnetic Resonance Imaging Techniques”) to probe the effect of acute ethanol intake on pregnant baboons and their fetuses. Specifically, the study examined the effect of ethanol on the inner layer of the uterine wall, known as the myometrium, and on fetal brain perfusion, which is a measure of cerebral blood flow (CBF) (Kochunov et al. 2010). The researchers measured brain perfusion before (baseline) and immediately following the administration of an ethanol dose equivalent to human binge drinking (see figure 1F). In the fetal brain, the peak contrast uptake concentrations and contrast uptake and washout rates were significantly increased after ethanol treatment, suggesting that the ethanol increases CBF. The researchers hypothesized that ethanol’s vasoactive properties are responsible for this CBF increase. This study also suggested that ethanol increased the permeability of placental membranes to the contrast agent, which is used to improve visibility of tissues during imaging. Specifically, the researchers found that more agent entered the fetal cerebral circulation, indicated by greater MR signal reduction in the fetal brain acutely following ethanol exposure. This is the first study to investigate ethanol’s effect on fetal CBF and placenta permeability using in utero DSC-MRI. The study suggests two potential teratogenic mechanisms of ethanol: ethanol-mediated changes in placental permeability and ethanol-induced changes in fetal CBF.

Although MR studies of nonhuman primate FASD models are sparse, a number of studies using invasive methods have been conducted to examine alcohol’s effect on the CNS in fetal ethanol exposed monkey fetuses. Multiple exposures of monkey fetuses to alcohol during specific developmental periods cause a reduced number of Purkinje cells in the cerebellum (Bonthius et al. 1996) and neurons in the frontal lobes (Burke et al. 2009). Two recent histological studies in fetal macaque monkeys found that an acute single exposure to alcohol during the third trimester causes widespread neuron apoptosis throughout gray matter regions (Farber et al. 2010) and glial cell (of the oligodentrocyte lineage) apoptosis across white matter regions (Creeley et al. 2013). These disruptions on a cellular level might contribute to the observed changes in neurometabolites observed in MRS studies in both human and animal FASD (Astley et al. 1995; Cortese et al. 2006; Fagerlund et al. 2006; O’Leary-Moore et al. 2008).

## Summary and Future Directions

The use of animal models in FASD studies has deepened our understanding of the biological bases of FASD, improving the accuracy of our interpretations of neuroimaging findings in human studies, and provided potential markers for future FASD diagnosis. A significant current goal of many research groups is the development of new noninvasive strategies for early detection of deleterious effects of prenatal ethanol exposure. As Streissguth and colleagues (2004) have noted, the odds ratios of several adverse life outcomes decrease in FASD individuals when therapeutic intervention strategies are initiated early in life. The rationale for this observation has been twofold: CNS plasticity decreases over the first few years of life (Olson et al. 2007), and early diagnosis of FASD is particularly important as it allows “capable caring families to advocate for their children’s needs (p. 235)” before establishment of maladaptive behavior (Streissguth et al. 2004). For these reasons, the design of methods for early detection of prenatal ethanol exposure-induced perturbation of normal development remains an important objective in applications of noninvasive neuroimaging tools and animal models of FASD.

As this overview has shown, many laboratories are engaged in research using animal models of FASD. They are implementing studies that vary the timing of ethanol exposure relative to CNS development, along with a diverse array of MRI modalities so they can better understand the consequences of ethanol exposure on anatomical, physiological, and metabolic development (see table). Among the advantages of using noninvasive neuroimaging techniques in animal models is the translatability of findings to clinical studies. In many cases, the biological bases of neuroimaging results obtained in human studies are not well understood. In these cases, parallel neuroimaging experiments with animal models can be performed, and interpretations of the findings can be validated with independent (but often invasive) experimental approaches. Efforts are being made to bridge the MRI findings to histopathological results, which are thought to be the gold standards in and outside FASD research (Jespersen et al. 2012; Leigland et al. 2013*b*; Riddle et al. 2011). In many other cases, studies using MR techniques to monitor CNS development and to examine CNS pathologies (other than FAS/FASD) also can provide valuable perspectives for FASD research. For example, ongoing diffusion MR microscopy efforts have been used to provide a detailed quantitative description of embryonic and early postnatal mouse brain development (Aggarwal et al. 2014; Zhang et al. 2003, 2006). Diffusion anisotropy maps derived from this method show excellent tissue contrast and, as a result, allow visualization of fine microstructural detail of the developing brain. This technique will be useful for investigation of ethanol-induced brain abnormalities in animal models over this age range. In addition, the development of advanced motion correction and imaging reconstruction technique has made in utero MRI examinations possible in humans as well as animals (Fogtmann et al. 2014). Using reconstructed in utero MRI, researchers can delineate human fetal brain tissues (including transient structures present only at early stages of development such as the cortical plate, intermediate zone, ventricular and subventricular zones, etc.) and can plot their growth trajectories (Scott et al. 2011). This close monitoring can help identify fetuses with growth patterns that deviate from the normal trajectory. In turn, their later cognitive outcome could be associated with growth patterns in future studies.

## Figures and Tables

**Table:** Findings of Magnetic Resonance (MR)-Based Fetal Alcohol Spectrum Disorder (FASD) Animal Studies.

**MR Modalities**	**MR Studies**	**Ethanol Exposure**	**Age at Assessment**	**Findings**
**Anatomical MRI**	Astley et al. 1995	Weekly, gestation week (GW) 1 to 3, or 1 to 6, or 1 to 24, 2 g/kg, intragastric gavage	2 to 4 years	No gross morphological abnormalities. No gross difference in size of cerebral hemispheres, corpus callosum, brain stem, or cerebellum.
	Monkey			
	Godin et al. 2010	Gestation day (GD) 7, 2.9 g/kg, intraperitoneal (i.p.) injection	GD 17	Holoprosencephacy (the forebrain fails to develop), cerebral cortical heterotopia (where clumps of gray matter develop in the wrong places), failure of the pituitary gland to develop (pituitary agenesis), dilation of the third ventricle.
	Mouse			
	Parnell et al. 2009	GD 8, 2.9 g/kg, i.p. injection	GD 17	Reduction of total brain volume. Comparison of individual regions revealed difference in all except the pituitary and septum.
	Mouse			
	Parnell et al. 2013	GD 9, 2.9 g/kg, i.p. injection	GD 17	Increase in septal region width, reduction in cerebellar volume, ventricular dilation, malformation of cerebral cortex, hippocampus and right striatum.
	Mouse			
	O’Leary-Moore et al. 2010	GD 10, 2.9 g/kg, i.p. injection	GD 17	Ventricular dilation, reduction in total brain volume as well as each of the assessed brain structures.
	Mouse			
	Parnell et al. 2014	GD 7 to11, 4.8 percent EtOH-containing diet (vol/vol)	GD 17	Decrease in cerebellar volume, increase in septal volume.
	Mouse	GD 12 to 16, 4.8 percent EtOH-containing diet (vol/vol)	GD 17	Reduction of right hippocampal volume, increase in pituitary volume.
	Leigland et al. 2013*a*	Daily, GD 1 to 20, 4.5 g/kg, intragastric gavage	PD 0, 3, 6, 11, 19, 60	Reduction of brain and isocortical volumes, reduction of isocortical surface area and thickness.
	Rat			
**Diffusion Tensor Imaging (DTI)**	Leigland et al. 2013*b*	Daily, GD 1 to 20, 4.5 g/kg, intragastric gavage	PD 0, 3, 6	Higher fraction anisotropy (FA) in cerebral cortex.
	Rat			
**Magnetic Resonance Spectroscopy (MRS)**	Astley et al. 1995	Weekly, GW 1 to 3, or 1 to 6, or 1 to 24, 2 g/kg, intragastric gavage	2 to 4 years	Increased Cho/Cr with increased duration of EtOH intake.
	Monkey			
	O’Leary-Moore et al. 2008	Daily, postnatal day (PD) 4 to 9, 5 g/kg intragastric gavage	PD 16	Increased NAA/Cr in cerebellum and striatum, Cho/Cr ratio was increased in striatum but decreased in hippocampus.
	Rat			
**Perfusion MRI**	Kochunov et al. 2010	GW 24, 3 g/kg, intragastric gavage	Immediately following ethanol exposure	Increased permeability of placental membrane, increased cerebral blood flow in fetal brain.
	Baboon			

## Notes

Glossary

Apoptosis: A process of programmed cell death.
Basal ganglia: A part of the brain located at the base of the forebrain and strongly interconnected with several brain regions, including the cerebral cortex, thalamus, and brainstem. The basal ganglia are associated with a variety of functions, including control of voluntary motor movements, procedural learning, and routine behaviors.
Caudate nucleus: One of three basic structures in the brain that make up the basal ganglia and is part of a system that is largely responsible for voluntary movement.
Cerebellum: The part of the brain important for motor control and other functions.
Corpus callosum: A wide, flat bundle of neuronal fibers that connect the left and right hemispheres of the brain.
Ex vivo: Experiments or measurements done on cells or tissues removed from an organism. Ex vivo conditions allow experimentation on cells or tissue under more controlled conditions than in vivo experiments.
Histopathology: The microscopic examination of tissue, indicating disease.
In vivo: Experiments or measurements done on an intact organism.
Morphology: The study of the form and structure of organisms and their specific structural features.
Thalamus: A part of the vertebrate brain made up of two halves deep in the middle of the brain. Among other things, it is involved in relaying sensory and motor signals to the cerebral cortex, and regulating consciousness, sleep, and alertness.
